# Typhus and Typhoid Fevers

**Published:** 1849-01

**Authors:** Joseph M. Smith

**Affiliations:** Prof. of Prac. Med. in Coll. Phys. and Surg., Chairman of Committee on Practical Med. A. M. A.


### SELECTIONS

FROM

### AMERICAN AND FOREIGN JOURNALS.

---

*Typhus and Typhoid Fevers.*—By Joseph M. Smith, M. D., Prof. of Prac. Med. in Coll. Phys. and Surg., Chairman of Committee on Practical Med. A. M. A.

Of all forms of infectious diseases, the one from which the greatest amount of mortality has occurred in most of the larger Atlantic cities of the North-eastern and Middle States, during the committee's term of service, is typhus, or, as it has been generally called, ship fever.

Though typhus is not perhaps as strictly entitled to the term epidemic as some other distempers, being never equally rife in the different classes of society, but mostly confined to persons dwelling in confined situations, in the midst of human filth yet its prevalence is sometimes so extensive, and its diffusion so clearly owing to the agency of a peculiar atmospheric influence, that there is no sufficient reason for not considering it as occurring epidemically. That it conforms to the law of periodicity, which determines the recurrence and spread of certain other maladies, is shown by its history in various countries. Pursuant to that law, though it may never be absent from a city or district, it increases at times, and after raging for a season, declines like other epidemics. Dr. Alison says, in speaking of the contagious fever of Edinburgh, that "there have been three great epidemics of that disease in the last twenty-two years, beginning in 18 7, 1826, and 1836, (the last of which has now nearly subsided,) each lasting nearly three years, and each of the last two affecting, he believes, nearly ten thousand persons." Of the typhus of Glasgow, Dr. Davidson remarks "that a severe epidemic of one or two years' duration is never succeeded by another until several years have elapsed." Similar facts illustrate the epidemic character of typhus in other European cities.

On examining the records of deaths from typhus for the last thirty-five years in the city of New York, it is found that the disease was epidemic in that period four times, viz: in 1818, 1827–28, 1837, and 1846–47. In these years the increase of the disease was so remarkable, that there is no hesitation in pronouncing them epidemic typhus periods. The fact now referred to was strikingly exemplified in 1837. In that year the mortality from typhus was 338, while that of the preceding years was but 117, and that of the following only 104. From this time, though the amount of mortality annually varied, there was no notable increase of deaths from typhus until 1846, when the number rose to 256; and the disease continuing to prevail and with increasing severity, the deaths in 1847 reached the frightful sum of 1396, to which should be added very many of 657 deaths from dysentery, a disease which in a large proportion of the cases in the hospitals, was typhus fever.

The foregoing facts fully warrant the inference that the diffusion of typhus is favored by a peculiar aerial influence which occurs periodically. Such an influence is sometimes very widely extended. It is said by Barker and Cheyne, in speaking of the epidemic typhus period of 1817–18, as quoted by Dr. Davidson, that "it must be acknowledged that the simultaneous increase of the disease in Ireland, and on the Continent, leads to the inference, that whatever may have been its origin, an epidemic constitution prevailed over a great part of Europe during a series of years past." Now such an epidemic constitution has manifestly prevailed over the British Isles during the years 1846–47; and its influence, in its western direction, appears to have extended across the Atlantic ocean to the shores of our North-eastern States and the adjacent British Provinces.

But coincident with this wide-spread epidemic meteoration, were other causes more immediately influential in predisposing to typhus on both sides of the Atlantic. The more efficient of these causes, especially in Ireland, were famine and the evils, physical and moral, attendant upon it. Whilst starvation and disease were destroying thousands of the poor in that ill-fated land, multitudes of them, to escape from calamities so fearful, sought to ameliorate their condition by a removal to this continent. In no annual period in the history of emigration, has the number of foreigners, added to our population, equaled that of the last year. It has been estimated that about a quarter of a million of emigrants arrived in the United States in 1847. Of this number, 160,134 landed at New York, and the remainder mostly at Boston, Philadelphia, Baltimore and New Orleans. Thousands also entered our country by the way of Canada and New Brunswick. A very large majority of those who landed at New York were Germans and Irish. It is stated by the New York commissioners of emigration, that of the 129,062 passengers arrived at that port, from May 5th to December 31st, 1847, 53,180 were from Germany, and 52,946 from Ireland, thus showing that the emigration from the former country exceeded that from the latter.

The condition of the German and Irish emigrants prior to their embarkation, and during their transit of the ocean, was in most instances conspicuously different. Whilst the former were generally robust, and well provided on the passage with the means of subsistence, and observant of cleanliness and ventilation,—the latter were in most cases enfeebled from want of sustenance, and on shipboard, destitute of supplies of wholesome food, depressed in mind, clothed in filthy garments, and crowded and confined in air rendered pestiferous by the excrementitious matters eliminated from their own bodies. In contrasting the hygienic circumstances in which the two classes of emigrants were placed, it is easy to account for the greater amount of sickness and mortality which occurred in one class than in the other. It is said, that of the admissions of emigrants into the hospitals and almshouse of New York, the Irish exceeded the German in the proportion of about one to nine or ten; and we are told, that the Irish in British ships suffered more than those in American.

The amount of disease and number of deaths which occurred in emigrant ships, while crossing the Atlantic, are appalling to contemplate. Many thousands perished on the voyage to the United States and Canada. In some ships bound to New York, from 20 to 30 died on the passage; and in many vessels destined to Canada, the deaths were from 3[illegible] to upwards of 100. From one ship, the Virginia, bound from Liverpool to Quebec, with 470 passengers, 158 of the number were buried at sea.

The Montreal Immigrant Committee, in their report for 1847, state, that "in no year since the conquest has Canada presented such fearful scenes of destitution and suffering." "The year 1847 has been unparalleled for the amount of immigration to Canada, near 100,000 souls have left the British isles for these provinces the past year,—over 5,000 of these died on their passage out, 3,389 at Grosse Isle, 1,137 at Quebec, 3,862 at Montreal, 130 at Lachine, and 39 at St. Johns, making in all at these several places 13,815. How many have died in other sections in Canada East cannot now be known, nor, indeed, how many have perished in Canada West; but coupling all those who have perished with those who have passed into the United States, Canada cannot now number 50,000 souls of the 90 odd thousands which landed upon our shores." In sketching a retrospect of these terrific scenes, the Montreal committee forcibly remark—"From Grosse Isle, the great charnel house for victimized humanity, up to Port Sarnia—along the borders of our magnificent river, upon the shores of Lake Ontario and Erie, and wherever the tide of immigration has extended, are to be found the final resting places of the sons and daughters of Erin—one unbroken chain of graves, where repose fathers and mothers, sisters and brothers, in one commingled heap, without a tear bedewing the soil, or stone to mark the spot. Twenty thousand and upward have gone to their graves, and the whole appears, to one not immediately interested, 'like a tale that is told.' "

The disease of which the emigrant passengers, and in many instances, the officers and crews of ships, perished at sea, and of which a great number were ill on their arrival in the United States and Canada, was typhus in its genuine form. In some ships dysentery, small-pox, and measles swelled the amount of mortality, and added to the number of sick that reached the ports of destination. From some ships upwards of 100 ill were landed at the New York quarantine station; and so great was the influx of sick and destitute emigrants, during the summer of the last year, that the public and private hospitals and almshouse were filled to overflowing, and sheds and tents were erected for their accommodation. In stating these things we cannot withhold the grateful remark that, in providing these accommodations, and furnishing other means of comfort, the same spirit of benevolence was manifested, which animated our countrymen in sending ships laden with supplies of food to the famishing in Ireland.

The number of persons admitted into the marine hospital at Staten Island, in the year 1847, was 6,932. Of this number, 5,277 were sick with fever, and 662 died. In that hospital, 2,229 cases were registered as typhus fever, of which 457 died, and 3,020 as remittent and typhus remittent fever, of which 205 died. There is reason to believe that many of these latter cases landed from emigrant ships, were typhus; but excluding these, the total number of deaths from typhus at the quarrantine hospital and within the city of New York, was scarcely less than 2,000.

But besides those admitted into the hospitals immediately after their arrival, or who sickened in the city and were sent to the hospitals, there were many who became victims of the disease at places, more or less distant in the countries over which they were dispersed.

The effect of introducing so great a multitude of persons affected with typhus, or imbued with the *fomes* of the disease, into the midst of the resident population, could not be otherwise than to excite alarm, and in fact, to extend the disease. Wherever the emigrant sick were congregated in masses, or collected in small numbers, or wherever individual cases were confined in small, unclean, and ill-ventilated apartments, the disease frequently attacked those who were in constant or occasional communication with them. From such communications nurses, physicans, friends, and transient visitors to the sick, have suffered in numerous instances. Of the medical officers in the New York and neighboring hospitals and almshouses not less, than eight, mostly in the vigor of early manhood, and while engaged in active duties, giving promise of future distinction in the ranks of the profession, have during the epidemic, been numbered with the dead.

But though the disease amongst residents has, in most instances, been traceable to exposure to the poison emanating from the persons and effects of emigrants, there is reason to believe that it has, in some instances, originated *de novo* in the close and sordid dwellings of the poor in our large sea-port towns; and that the mortality from the diease hasbeen augmented from such sources. This statement derives support from the circumstance, that the diseases prevalent in our nothern Atlantic cities for the last two or three years, and especially in New York, have manifested a decided adynamic diathesis, a circumstance indicating it is believed, the existence of a meteoratious influence, which not only develops a predisposition to typhus, but favors the production of the poison of the disease.

As to the mode in which the late typhus, or ship fever, has been propagated, the committee are not disposed to enter into an elaborate disquisition. The question whether typhus is diffused by a specific contagious principle, or by a vitiated human effluvium, may be regarded as theoretical, and therefore affords grounds for a diversity of opinion. The fact that patients affected with the disease, in certain conditions, are *foci* of the typhus poison, must be granted by all observers. But the admission of this fact does not necessarily impose the obligation to consider the poison a specific *virus*, analagous in its origin and distinctive properties to that of small-pox or measles, and that it is not so, is the view taken of it in this report. As here contemplated, it is the product of the chemical changes which take place in the excretions or *debris* of the body in health and disease, accumulated in close apartments,—a poison more frequently and readily generated by the exhalations and other excretive matters of typhus patients, than by those of persons suffering under other forms of the disease, or in the state of health,—a subtle aëriform principle, appropriately denominated *idio-miasma*, one of the generic forms of infection; and hence, typhus, instead of being classed among the *contagious* epidemics, is arranged among the infectious. In this theory is found a ready explanation of the origin and prevalence of typhus in jails, hospitals, ships, and the houses of the poor; and also, of the mode in which cleanliness and ventilation arrest and exterminate the disease. These hygienic means, so effective against the general propagation of the disease in the upper classes of society, seem clearly to intimate the true nature and source of the typhus poison.

So entirely were the symptoms and course of the ship fever, as it occurred in New York, correspondent with the descriptions given of typhus in the standard works on Practical Medicine, that nothing in detail need be said in respect to them. It is sufficient to observe, that among the emigrants the malady was marked by great prostration at an early period of its development, requiring liberal administrations of the most efficient means of stimulation and support. Petechiæ were more general than in some former years. Though some times running to a fatal issue under a simple form, the disease in its graver varieties, was generally attended with cerebral. thoracic, or abdominal complications. Erysipelas, often assuming a phlegmonous character, occurred in many cases, and frequently resulted in extensive sero-purulent deposits.—Fluid collections of this kind were most common in the parotid glands, eye-lids, and scalp. Secondary attacks happened in a few instances; and among the sequelæ was noticed an affection of the eyes of an amaurotic form.

As to the rate of mortality among the sick in the various hospitals of the United States and of Canada, there has been a great disparity; the differences depending on circumstances, in many cases, beyond the reach of control. In the New York hospital, as it is stated in the last annual report of the governors of that institution, "the number of typhus fever cases treated during the year, was 1,034; very many of them cases of the severest form of the disease, and demanding far more than the ordinary assistance of physicians and nurses. Of this number 136, or a little more than thirteen in the hundred, have died. This result, though it appears to be a large proportion, bears a very favorable comparison with the statistics of any other sanitary establishment under similar circumstances of disease, and affords a very striking contrast to the terrible mortality from the same cause among the recent emigrants from Europe in other parts of the continent." But though the amount of mortality has been influenced by the circumstances in which the sick in hospitals were placed, it is to be noticed that the proportion of deaths has been greater among those in the higher than among those in the lower ranks of life, a fact generally observed in the typhus epidemics of Europe.

With these remarks on the typhus or ship fever of the last year, we might close our report, were it not that a question of more than ordinary interest, relating to this subject, demands from us a careful examination,—a question on which the profession in this country and in Europe are divided, and which, it is thought, the phenomena of the recent typhus epidemic, added to the facts afforded by former prevalences of the disease, have largely contributed to elucidate. It needs hardly to be stated that we refer to the question of the identity or non-identity of typhus, and a form of disease entitled typhoid fever. That there are two forms of fever, bearing these names, which closely resemble each other, and yet which are essentially distinct in their nature, is alleged by some of the most renowned pathologists of the age. The leading distinctive features of the two diseases are, it is affirmed, intumescence and generally ulceration of Peyer's glands, and certain other morbid conditions, in one of them, and the total absence of these phenomena in the other.

Now if, in fact, two such diseases exist in nature, the committee, in what they have submitted concerning epidemic typhus, have omitted to distinguish them, or, in other words, have committed an error in confounding distempers specifically dissimilar; for everywhere have the two forms of disease been associated and looked upon as one and the same malady. The attempt to rectify such an error, if such have been made, would involve the necessity of giving the history of two epidemics, occurring cotemporaneously in the same localities, one to bear the title of *typhus*, and the other of *typhoid fever*, and of sustaining the distinction between them,—a labor not achievable in the hands of your committee.

But it is believed that no such distinctive disorders can be recognized in nature; that the two forms of disease have not respectively a development which is peculiar or *sui generis;* that the disorders in question do not, as distinct specific affections, occur promiscuously, or side by side, in the same circumstances, from the same causes, among the same class of persons, in the same hospitals, ships, jails; and private dwellings of the poor. The grounds on which we venture to express these opinions we beg leave respectfully to state to the Association; and we do this with the more freedom, as the question of the identity or non-identity of typhus and typhoid fever should and will continue to attract attention until a unanimity in regard to it is attained among the great body of enlightened physicians.

As the forms of disease in question are fevers, and as the problem to be solved is, whether they are, or are not, one and the same malady, it will not be improper to glance at the means by which fevers are distinguished. It is much to be deplored that the distempers comprised in the class *febres* have not been more perfectly disentangled and their specific relations to one another definitively settled. That this has not been done, is owing, not to any want of labor or talent devoted to the work, but to the difficulties and obscurities inherent in the subject.

The means of diagnosis in fevers are threefold; 1st, their efficient causes; 2dly their symptoms; and 3dly, the alterations produced in the organism discovered after death.

In regard to the efficient causes of fever, it is evident that a knowledge of them, for the purpose of diagnosis, is of the highest value; for were they in all cases ascertainable, they would furnish not only a ready and correct diagnosis, but a solid basis on which to rest a natural classification of fevers.

In respect to the symptoms, in the absence of a knowledge of the causes of fevers, it is clear that they are the only means by which, during life, one kind of fever can be distinguished from another. But in forming a diagnosis by these means, the mode of proceeding is in some respects peculiar. Whilst the acute phlegmasiæ and most other violent local diseases are generally cognizable, by their rational and physical phenomena, immediately, or at an early period after their development, it is frequently otherwise in the case of essential fevers. These diseases are not so commonly distinguishable at first sight. In order to collect the facts from which their differential diagnosis may be deduced, it is often necessary to observe them carefully, sometimes in more than single cases, during the space of several successive days. Moreover in the phlegmasiæ, the diagnostic phenomena are for the most part local, being confined to the organs immediately affected. In fevers it is not so; the diagnostic signs are spread throughout the economy, and are slowly and successively developed, and not until a certain number or variety of them have come fully into view, can a specific fever be recognized.

Of the anatomical alterations produced by fevers, it may be said that such of them as occur in the internal organs, and which are discoverable only after death, have no absolute claim to the character of diagnostic phenomena, since none of them are invariably present in the same specific fever and many of them are found in different kinds of fever. There are, however, in some fevers, certain alterations in the organism so frequently revealed by autopsic examinations, that they may not improperly be admitted among the accessory means of diagnosis, especially in cases in which neither the causes nor the symptoms during life are sufficient to elucidate their distinctive nature.

From these general observations, we proceed to the question of the identity or non-identity of typhus and typhoid fever. And in doing so, leaving out of view for the present the history of their causes, we shall inquire:

1st. Whether these diseases differ so much in their symptomatology as to enable us to characterize them as distinct distempers?

2dly. Whether the morbid anatomy, and particularly the intestinal follicular lesions observed after death, indicate the diseases to be specifically different?

1. *Of the Symptomatology of Typhus and Typhoid Fever.*—In essential fevers there are a few symptoms which are common to them all; and to these symptoms are added, in particular fevers, certain phenomena which arise in the progress of the morbid movements that mark their special form and character. Now among the leading symptoms, mostly of the latter description, observable in typhus and typhoid fever, are the following: great prostration of the sensorial and muscular energies, cephalalgia, disagreeable dreams, somnolence, delirium, coma-vigil, deafness, aphonia, stupor, subsultus tendinum, picking of the bedclothes, and at imaginary objects in the air, small, frequent and feeble pulse, pungent heat of skin, petechial eruptions, sudamina, dry and brown or black fur on the tongue, accumulations of dark sordes on the teeth and gums, epigastic pain, tenderness of the abdomen, gurgling in the intestines, diarrhœa, meteorism, hemorrhage from the nose and bowels, and retention of urine.

Though such are the phenomena which occur in typhus and typhoid fever, yet all of them may not present themselves in every case; but it is observable that many of them, and especially those attendant on the last stage of fatal cases, are developed consecutively and in various degrees of force, in every individual example of both diseases.

Now it is among the phenomena enumerated, that we must seek for the diagnostic symptoms of the two diseases, if any such phenomena exist. If the diseases be specifically different, they should respectively present certain pathognomonic or characteristic symptoms. If they possess no such symptoms, nor any other diagnostic phenomena, they are obviously not distinct disorders. But let us examine the symptoms with a view to ascertain how far they indicate diseases different in their nature.

It is generally admitted that there is much variety in the symptoms of the premonitory and successive stages of typhus; and a similar variety, it is acknowledged, occurs in the corresponding stages of what is called typhoid fever. The incipient symptoms are often, for the most part, precisely the same in both maladies; and in whatever subsequent parallel stages we examine the two disorders, we find no signs by which to distinguish them as different species of fever. If in the several periods or stages of typhus we observe particular combinations of symptoms, we find the same combinations in the several periods of typhoid fever. Phenomena, which are usually qresent in typhus, are sometimes absent, or but slightly developed; and the same is true in typhoid fever. The constitutional phenomena in the latter disease are grouped, and transformed in the same manner as are those which occur in the former. So far then as the general symptomatology of these diseases enables us to form a judgment, they are essentially the same. This conclusion, it is believed, might be sustained by an appeal to numerous cases recorded as genuine examples of typhus and typhoid fever.

The phenomena on which the advocates for the distinctive nature of typhoid fever mainly rely in forming their diagnosis, are, it appears to us, totally insufficient to establish the specific character of the disease. The more important of these phenomena are lenticular rose-colored spots on the trunk, chiefly on the anterior part of the abdomen and chest, sudamina, gurgling in the right iliac region, tenderness of the abdomen, diarrhœa, meteorism, enlargement of the spleen and epistaxis. But which of these phenomena does not occur in typhus? In regard to the size, color, situation, and the time of appearance of the eruption, there is nothing remarkable in the one disease which is not equally so in the other. The eruption in typhus, though among the characteristics of the disorder, is variable in size and color; sometimes it is of a rose hue, but frequently violet or purple, at one time persistent under pressure, and at another evanescent. Such were the varieties of the eruption in the cases of typhus, treated under the direction of one of your committee, in the New York Hospital in June, 1840, and at various times in subsequent years, cases of the identical nature of which there was not the slightest doubt, all of them having originated from the same source; namely, the fever poison *idio-miasma*, generated in crowded emigrant ships. In no respect did the eruption differ essentially from that which occurs in dothinenteritis or typhoid fever. As to sudamina, they are common to various states and kinds of disease.

It is well known that age, constitution and modes of life, climate, season, and especially epidemic influences exert a powerful agency in modifying the symptoms of diseases; and there is good reason to believe that to such causes, the varying forms and character of the eruptions in essential fevers are in a great degree attributable. In some seasons the eruption in typhus is absent in very many cases; and moreover, it is sometimes wanting in cases in which the Peyerian glands are extensively diseased. Dr. Elliott, one of the physicians of the Bellevue Hospital, reports an instance in which "Peyer's plate were much enlarged and deeply ulcerated," and in which "there was no eruption, nor any diarrhœa, during the whole progress of the case. Are not the rose-colored spots of typhoid fever the primary forms or conditions of the petchiæ or maculæ of typhus? Prof. Clark, one of the physicians of the hospital just named, says, that "in the proportion of about one-half of the typhus fever patients, a rose-colored eruption occurs on the body, which frequently in the progress of the fever assumes a petechial appearance." To elevate the shades ofcolor, and the varieties of form of the eruption to the rank of diagnostic phenomena, indicative of distinct diseases, when the more striking and important phenomena are unequivocally expressive of an identity of disease, is a refinement which it appears to us, no clinical observer can regard with favor.

Nor are the diarrhœa and gurgling in the right iliac region worthy of consideration, as signs by which the affection called typhoid fever may be distinguished as a specific disease, or one different from typhus. These phenomena occur in cases in which the general symptoms of the two forms of disease, arising from disturbance of the nervous, vascular, and secretory functions, are the same and which therefore indicate the affections to be one and the same. Gurgling in the bowels is common to many diseases; and as to diarrhœa it frequently occurs in fevers in which there is no follicular disorder of the intestines; and though it is generally present in dothinenteritis, it is sometimes absent. Dr. Swett, late President of the N. Y. Medical and Surgical Society, so well known for his devotion to pathological studies, in discoursing on the cases of ship fever, treated at the New York Hospital, at a meeting of that society on May 1st, 1847, stated that "in the case in which the ulceration was most extensive, there had been no diarrhœa; in the other case with ulceration less in amount, though still considerable, the diarrhœa had been profuse." Similar remarks are applicable to the meteorism and tenderness of the bowels; they frequently occur in disorders different in their character, and are occasionally wanting in typhus and typhoid fever. With respect to epistaxis and delirium, they are totally destitute of any claim to the character of signs denoting a specific difference between the two forms of disease in question.

But though the rose-colored spots, the diarrhœa, and gurgling in the bowels, the meteorism, tenderness of the abdomen, epistaxis, and delirium, taken severally, or any two or three of these phenomena combined, do not enable us to distinguish typhus fever from the typhoid affection of Louis, still it may be asked, do they not, when collectively present, that is, when grouped together in the same case, indicate these diseases to be essentially different? In regard to this question, it is to be observed, that in discriminating fevers, the ablest clinical physicians place more reliance upon the uniform constitutional phenomena, as manifested in the morbid conditions of the nervous, vascular, and secretory organs, than upon those phenomena which are local and contingent. The former are esteemed the efficient, and the latter the accessory means of diagnosis. Are not intermittent and remittent fevers known and distinguished by their types and general phenomena, and particularly by the modes in which the phenomena are combined or succeed each other? In these forms of fever there is often inflammation or engorgement of particular organs, and yet, notwithstanding such complications, each of these diseases is readily recognized and distinguished. Even in yellow fever, the course and form of the general symptoms, springing from derangement of the nervous and vascular systems, are vastly more important as indications of the specific nature and distinctive character of the disease, than the symptoms immediately connected with the gastro-intestinal lesions. These latter, it is true, are sometimes valuable subsidiary means of diagnosis; and, indeed are occasionally essential aids in deciding in doubtful cases; but aside from the general phenomena, they would scarcely suggest the idea of yellow fever; whereas, the constitutional symptoms are, of themselves, sufficient, especially when the disease is epidemic, to show the true character of the malady. The same remarks, in regard to the symptoms depending on special organic lesions, are applicable to every other essential fever. In the contagious exanthemata it is otherwise. In these, the eruption is, with very few or no exceptions, a constant occurrence, and is therefore regarded as one of their leading diagnostic phenomena.

Now if we examine the phenomena of typhus as described by English writers, and compare them with those of typhoid fever as described by M. Louis and others, are we not warranted in giving to the general symptoms a higher value as diagnostics of the special nature of these diseases, than to those which indicate an affection of the glands of Peyer? Is not typhus distinguished in all cases by the character and train of the general symptoms, rather than by any disturbance of the abdominal organs, indicated by tenderness under pressure, diarrhœa, gurgling in the right iliac region and meteorism? And is not the same true of typhoid fever? That such is the fact in respect to the latter disease, there can be no question; for Louis and others have recorded cases as typhoid fever in which the symptoms of follicular disease of the bowels were obscure or not conspicuous.

Idiopathic fevers, then, are distinguished by the kind and character of their general symptoms, and by the mode in which these are grouped and successively developed. In comparing fevers with one another, the order of the occurrence, as well as the peculiarity of all the phenomena, should be observed. When this is carefully done, there is generally no difficulty in discriminating particular fevers. All cases of fever are to be considered of the same nature in which the constitutional symptoms occur in the same order, and agree in their general features. The local, contingent, and minor symptoms, as well as the variations in the mode of attack, violence and duration of the morbid phenomena, indicate modifications of the disease, and nothing more. A physician practically versed in the diagnos s of fevers, on visiting the wards of a hospital in which the various species of essential feversar e assembled, will distinguish each particular kind of fever, not by tenderness of the abdomen, gurgling in the bowels, diarrhœa, and epistaxis—for these phenomena may or may not be present in fevers unlike each other—he will found his diagnosis on the type, and general course and peculiarity of the phenomena manifested in the nervous vascular, and secretory functions. In individual cases, in which the type and characteristic symptoms of a special form of fever are not as fully and clearly developed as is necessary to settle the diagnosis, he will await their occurrence, inquire into the etiology of the disease, and avail himself of the light thrown on the nature of the malady by the occasional or contingent symptoms. Thus, in yellow fever—a disease usually well marked and distinguishable by a train of general symptoms, cases occur in respect to which a doubt may exist as to their true nature, until the appearance of black vomit, an event in itself of no value as a diagnostic of a specific form of fever, except when it concurs with certain diagnostics presented by the general system.

Now in typhus and the typhoid fever of Louis, the morbid phenomena are so correspondent in the sensorial. vascular and other functions, and are so similarly catenated or related to one another from their accession to their, termination, that the attempt to assort and group them, so as to make them express two different diseases, is a labor which it appears to us, must fail to show any distinction which is found in nature and cognizable by practical minds.

If such be a just view of the subject, in what light are we to regard the phenomena considered by Louis and others, as establishing a specific difference between the two forms of disease in question? To say nothing of the rose colored spots, delirium and epistaxis, are not the meteorism, tenderness of the abdomen, gurgling in the intestines and diarrhœa, occurring in fever, simply indicative of abdominal derangement, in the same manner as cough expectoration, and certain auscultatory signs denote a pulmonary complication, or as injection of the adnata, intolerance of light, headache and delirium, show encephalic disorder? The principal anatomical lesions in typhus are found in the three great cavities; and there are no reasons that would warrant the forming of a special disease of those cases in which abdominal symptoms are prominently developed, which would not equally justify the creation of new diseases of cases in which pneumonic and cerebral symptoms are predominant. If the term abdominal typhus, applied by the German physicians to dothinenteritis, be appropriate, it may not be improper to apply the terms *thoracic* and *cerebral* typhus to those varieties of the disease in which the lungs and brain are the organs that chiefly suffer organic changes. These terms, so used, import special complications of typhus, but not distinct diseases.

That every case described by Louis as typhoid fever was genuine typhus is, perhaps, doubtful; but that most of his cases in which he found, after death, follicular ulceration of the bowels were not that disease, he has, we think, failed to show by any diagnostic phenomena. Had no autopsies been made, the cases would have been considered, by every pathologist, as simple modifications of the typhus of England and other countries. In searching for novelties in pathological anatomy, it should be borne in mind that the evidence for and against the theory of the essential nature of fever vastly preponderates on the side of the former; and consequently, all anatomical lesions are to be considered contingent or secondary. The anatomical alterations in typhus and other kinds of fever sometimes vary so much in different cases, that were one called upon to decide on the nature of the disease in a particular case, by inspection of the body after death, it would frequently be difficult, and sometimes impossible to say of what species of fever the patient died. Now such an embarrassment, in forming a diagnosis, rarely occurs in cases in which the symptoms, during the normal course of the disease, are carefully observed. In the phlegmasiæ proper, a post-mortem diagnosis is in general, not left in doubt, but is promptly and certainly attained.

Upon the whole, it seems to us, that the ingenious attempts which have been made to establish a specific distinction between typhus and typhoid fever, by an analysis of the symptoms, have yielded no other profitable result than a lucid exhibition of the modifications which genuine typhus assumes under different circumstances of temperament, habits and mode of life, climate, &c. In no other light can we regard the able researches on the subject by Louis and numerous other pathologists in Europe and America.

2. *Of the Morbid anatomy of Typhus and Typhoid Fever.*—As, then, the vital phenomena, observed during the progress of the two forms of disease in question, lead to the conclusion that they are not specifically distinct in their nature, it seems that the opinion that they are so, has arisen from the discovery of dothinenteric lesions in one form of the malady and not in the other. The theory which localizes the cause of fever in the stomach and intestines, so fashionable in the last thirty years, is obviously the source to which may be traced the distinction which is made between typhus and typhoid fever. Stated in a brief form, the theory, in relation to these diseases, is explicitly this; a fever with ataxic or adynamic symptoms in which the glands of Peyer and Brunner are tumefied and ulcerated is typhoid fever; and that a fever attended with similar constitutional phenomena, and in which those glands are not diseased, is not typhoid, but some other species of fever. That Louis employs the fact of the non-existence of dothinenteric lesions in febrile cases, to prove that the disease is not typhoid fever is shown in his work on yellow fever, in giving the diagnosis of which he specially distinguishes the two diseases anatomically, by the normal condition of the intestinal glands in one of them, and the constant alteration of them in the other.

That there is a great and fundamental error in the doctrine which makes a specific difference between the diseases to which our inquiries relate, may, we think, be shown by adverting to a few of the anatomical facts and general arguments which bear directly on the subject.

If a diseased state of the agminated isolated glands of the bowels constitute the peculiar and essential anatomical character of typhoid fever, then certain other maladies considered by every observer as different in their nature, should be regarded as identical with that disease, for there is abundant evidence to show that in many instances of the disorders referred to, those glands are inflamed, tumefied or ulcerated.

It must here be noticed that by the term anatomical character of a disease is meant, according to Louis, a morbid condition of organic lesion which invariably occurs in the same disease, and which, with certain other phenomena, distinguishes the disease from all others. Thus, in speaking of yellow fever, he says: "The red or black matter found in the stomach and intestines, not having been found in all the cases, it cannot be considered an anatomical character of that disease." But he adds, "it is not so with the alteration of the liver, which was more or less exactly the same in all the cases, and which for that reason ought to be considered as the essential anatomical character of the yellow fever of Gibraltar of 1828."

Now, if we use the term anatomical character in the rigorous sense in which it is employed by Louis, are we not bound to consider every fever in which Peyer's glands are diseased or ulcerated as typhoid fever? No special form of lesion of the intestinal glands, analagous to the special form of lesion of the skin in small-pox, is described as distinctive of the typhoid affection, for every morbid condition of them, except perhaps the tuberculous, is recognized in the disease.

The truth is, disease of the glands of Peyer and Brunner is, in none of its forms, in the strict sense of Louis, an anatomical character of any one species of fever. It occurs in disorders unlike in their causes and dissimilar in their character. It occurs occasionally in the different kinds of *koino-miasmatic* fevers, and also, now and then, in the contagious exanthemata. It occurs especially and most frequently in *idio-miasmatic* or genuine typhus, and in febrile maladies which, in their progress, become adynamic, or, as it is usually and properly said, typhoid. It occurs more generally in typhus, in some years, and in some localities and countries than in others; and when it occurs it is an effect or complication, and not the cause of the febrile disturbance of the system.

If these statements be correct, and that they are so, might, we think, be established by abundant testimony, there are no solid reasons for considering the affection of Peyer's glands a feature distinguishing the typhoid fever of Louis from the typhus of England and other countries. We have already shown that these diseases are so similar in their symptoms, that they cannot by these means be rationally characterized as different species. Whatever variety of forms they exhibit in their general or local phenomena, they scarcely ever fail to manifest a unity of type and character; and consequently they should be regarded as the same disease. Indeed, as we have said before, were it not that post-mortem examinations had revealed a lesion of Peyer's glands, there never would have been a question as to their identity.

With all the light which modern pathological anatomy has shed on the nature and complications of fever, and with all the aid afforded by researches directed to the elucidation of the connections between the symptoms of fevers, and the structural alterations which occur in this class of diseases, can it be determined with a degree of certainty, approaching the exactness with which we are able to announce the existence of a pulmonary or cerebral complexion, that the glands of Peyer are diseased in a given case of fever? A high degree of probability that they are affected may be inferred from particular symptoms; but after all, nothing but an autopsy can establish the fact.

That dothinenteritis occurs in genuine typhus is proved by the concurrent testimony of many physicians, who have enjoyed the most extensive opportunities of studying the disease, in all its forms and varieties of complication, and whose learning and talent for observation entitle their opinions to implicit confidence. While on the one hand, it is shown by their inquiries that the intestinal glands are frequently affected in typhus, it is, on the other, demonstrated that in the form of fever denominated typhoid, the follicular disease of the bowels is sometimes wanting. To cite all the proofs which might be adduced in support of these remarks, would require wider limits than the present occasion affords. The statement of a few facts, bearing on the points in question, will sustain what a greater nnmber could not render more evident. And first, let us advert to the evidence of some European authorities.

In no country is typhus more prevalent than in Ireland. The disease is there seen in all its varieties, symptomatic and anatomical; and it is to the enlightened physicians of that country that we are indebted for much valuable information in relation to the nature of the disease. Dr. Kennedy, in giving an account of the typhus which prevailed in Dublin in 1837–38, says: "The only post-mortem appearance that was at all constant, was congestion of the vessels of the membranes of the brain." "But if this epidemic typhus was not characterized by any uniform or constant pathological changes, it was very remarkable for the almost total absence of those abdominal lesions which have been regarded by some as the invariable attendants of typhus fever. Indeed, in this respect, this epidemic affords a striking and conclusive refutation of the false and hasty generalizations of the French pathologists on this subject. In this respect, also, it differs altogether from the epidemic fever of 1826, (which the writer had an ample opportunity of investigating while in charge of fever patients, supported at the expense of the government, at the Meath hospital that year,) in which in a large proportion of the post-mortem examinations made by him, more or less disease of the glands of Peyer was found." The typhus epidemics here brought into contrast by Dr. Kennedy, happily illustrate a well-known law of epidemics, namely, that the same popular disease varies in its form and anatomical lesions in different years and seasons. There can be no question that the two epidemics of which he speaks were essentially the same in their nature, and the difference in the condition of the intestinal canal in the two epidemics was incidental, certain influences in 1826 operating to produce "in a large proportion" of the cases, "more or less disease of the glands of Peyer," whereas, in 1837–38, the analogous modifying influences occasioned scarcely any organic changes in the bowels, but chiefly a different complication, "a congestion of the vessels of the membranes of the brain."

Dr. M'Cormac, of Belfast, who evinces in his work on fever great familiarity with the diseases of Ireland, after minutely describing the various changes which take place in the aggregated and solitary follicles of the intestines, remarks: "This exanthem, if we may call it so, does not present the regular phases seen in small-pox; it may exist during the greater portion of the disease, and even after the fever has terminated." And he adds. "after what I have said, I need hardly repeat again, that together with its results, it is on by an occasional contingency in fever, and seemingly more frequent on the continent than in this country—from what cause, however, if it be this, I do not pretend to say."

Dr. Stokes, of Dublin, than whom there are few physicians more capable of arriving at correct conclusions on pathological subjects, regards "typhus as an essential fever which affects in an especial manner in different cases, and during different epidemics, either the head, the chest, or the abdomen." He alleges that "the ulcerations of the intestines so much insisted upon by the French pathologists, are neither of constant occurrenee, nor are they characteristic of the disease; in one epidemic they are found on dissection, much more frequently than in another." Dr. Graves, the able colleague of Dr. Stokes at the Meath Hospital, entertains the same view of the nature of typhus.

In accordance with these opinions, are the extensive observations made in England, recorded in the writings of Drs Hewet, Tweedie, Copland, Southward, Smith, Marshall Hall and others. All these concur that the glands of Peyer are frequently the seat of anatomical changes in typhus.

Similar investigations in Scotland have established the same facts in regard to the pathological anatomy of the disease in that country. Dr. Davidson, in his Thackeray Prize Essay on typhus fever, gives a table showing the number and kinds of lesions observed in the post-mortem examinations of sixty-three eruptive cases admitted into the Glasgow Fever Hospital, from May 1st to November 1st, 1839, from which it appears that enlargement of Peyer's glands and ulceration of the intestines were present in many cases.

These are but a few of the evidences of the occurrence of dothinenteric lesions in the typhus of Great Britain and Ireland. But it may be said that the pathologists of these countries confound diseases which are specifically different; and that this is owing to their mode of studying the phenomena not being so minute and philosophical as the method pursued by the physicians of France. If the French make distinctions where the English do not, are we to accord to the former superior powers of discrimination? The English, Scotch, and Irish physicians have given evidence of talent and industry in the investigation of febrile pathology not surpassed, we think, by the medical men of any country. They have patiently examined the grounds upon which their continental brethren have drawn a line of distinction between typhus and typhoid fever; and having done so, they deny that there is any foundation in nature for it. Dr. Davidson, who, it is believed, expresses the general opinion of British physicians, says: "Would it not therefore, be refining our classification beyond all precedent, to separate typhus and typhoid fever with two species, where it has been shown that the symptoms in both are the same, or very nearly so, that they have nearly the same laws, as far as these have been ascertained that the severity of the symptoms in both is not in proportion to the lesions of the intestinal follicles; and that the other complicications of both are similar, although various in the same place at different periods, while the only characteristic in dispute has been acknowledged not a constant, and therefore, not a necessary element for the existence of the disease."

Such, then, being the opinions of physicians practising in the British Islands, it becomes a question of curious interest, if nothing more, to learn what impressions have been produced on the minds of those who, conversant with the typhoid fever of Paris and other continental cities, have personally compared this disease with the British typhus. Dr. Lombard, of Geneva, so often quoted on this subject, during his visit to England, Scotland, and Ireland in 1836, examined several cases of typhus, two in Dublin, and one in Glasgow, and finding the symptoms similar to those of the typhoid fever of the continent, with which he was familiar, expected to find after death the usual dothinenteric lesions; but on examination, to his great astonishment, no such lesions were found. He says, "in the whole course of my experience I have met with nothing which has surprised me more than this occurrence; I had been for years engaged in the study of typhus fever, and for years my almost daily experience in the dead-room led me to associate certain lesions of the alimentary canal with the symptoms of this disease, when suddenly I find myfelf assailed by a new experience exactly contradictory of my former; nor was my new experience unconfirmed by that of the Glasgow or Dublin physicians." With such facts before him, to the question, "whether the two diseases are different or the same?" he answers: "I cannot allow that they are specifically distinct, and consequently I am almost forced to give up the opinion that the local changes of structure are of paramount importance in causing or producing the symptoms that accompany this type of fever." Though Dr. Lombard subsequently resumed the opinion that the two diseases are different, it appears to us he did so without sufficient reasons.

In the following year, 1837, Dr. Staberóh, of Berlin, made a visit to Great Britian and Ireland, and for six months devoted himself to the study of the fevers of those countries. His intimate acquaintance with the typhoid fever of Paris especially qualified him to compare the diseases in question; and, after doing so, he concluded, that though in many cases the British typhus wanted the follicular lesion of the bowels, the disease was the same as the typhoid affection of the continent.

To the above authorities in favor of the identity of the two forms of disease, we must not omit to add that of M. de Claubry, whose able and elaborate investigations of the subject were published in Paris in 1844. His assemblage of facts, and the inferences he draws from them, seem scarcely to admit of being rationally controverted.

It thus appears that there are many of the more eminent pathologists of Great Britain, Ireland, and other European countries who concur in the opinion that no essential difference exists between typhus and typhoid fever. Their observations, however, establish the fact, that dothinenteritis frequently modifies the character of typhus, and that this complication is more common in some seasons and some countries that in others—a circumstance referable to causes which, though obscure, are clearly adventitious.

The necroscopic researches in this country have afforded no satisfactory evidence of there being a specifiic difference between the forms of malady under consideration. On the contrary, the facts already spread before the public, as well as those daily accumulating in our hospital registers, when carefully analyzed and compared with the phenomena of the typhus and typhoid epidemics of other countries, show that these diseases are one and the same. Cases presenting the same general features, the same nervous, vascular, eruptive, and other phenomena, and springing from the same efficient cause, are found to differ merely in their complications, one case exhibiting after death various anatomical changes, particularly alterations of the glands of Peyer and Brunner, and others showing no such alterations.

The strongest testimony, as it appears to us, yet adduced on this side of the Atlantic in favor of the opinion of the non-identity of typhus and typhoid fever, is that furnished by Dr. Gerhard of philadelphia. This gentleman has described, in the American Journal of the Medical Sciences for 1837, two epidemics, occurring in that city in different years, one of which he designated typhus and the other typhoid fever. In speaking of the former (typhus), he says, "in this large number of autopsies, amounting to about fifty, there was but in one case, and that doubtful in its diagnosis, the slightest deviation from the natural appearance of the glands of Peyer." In the epidemic which he denominated typhoid fever, it appears that Peyer's glands were generally affected, and hence he inferred that, notwithstanding the similarity of the general symptoms to those of typhus, the malady was not typhus. Now, if what has been said of the variety of complications which the same epidemic disease assumes in different years, be true, is it not fair to conclude that the differences in the morbid anatomy of the two epidemics described by Dr. Gerhard, were due to incidental causes; and that the two epidemics were the same disease modified in different seasons, as in the instances mentioned by Dr. Kennedy and adverted to by Drs. Stokes and Davidson?

The post-mortem examinations made at the New York Hospital, during the last year, might be deemed sufficient, apart from all other testimony, to establish the identity of typhus and typhoid fever. In the cases of ship fever, a pure form of typhus, a disease unquestionably originating in every instance from the same poison, follicular disease of the intestines was found after death in some cases, and not in others. Dr. Stone tells us, in the New York Annalist, that, in four weeks ending May 12th, 1847, there were admitted into the Bellevue Hospital about four hundred and sixty-six cases, sick of typhus; and he says that "the most constant anatomical lesion which I have found in the examination of about twenty-five of those who have died, is an enlargement and softening of the spleen. Peyer's glands were found more or less diseased in about one-fourth of these cases, and the heart more or less softened in a somewhat larger proportion." The same gentleman, in another communication, published in the New York Journal of Medicine, relating to the same fever, gives the following among his other conclusions, drawn from observations of the disease in New York and New England: "That typhus and typhoid fever, so called, are identical." Professor Clark, in illustrating the morbid anatomy of typhus, at a meeting of the New York Pathological Society, Feb. 23, 1848, stated, in reference to a particular hospital case, that "the usual old typhoid lesion affected the intestines with the common appearance of the present epidemic." "This case," he said, "allies the present epidemic with the typhoid fever." At a meeting of the same society held March 8, 1848, Dr. Swett made the following interesting statement. He said, "that fever had occurred at the Bloomingdale Lunatic Asylum, carried thither by an insane emigrant and fifteen persons had been attacked with it. Nine or ten had come to the hospital, and two had been examined post-mortem. In the first there were no lesions of any kind; in the second there were ulcerations of the small intestines." "Where the ulcers existed, instead of attacking Peyer's glands in the middle, they attacked the edges, extending two or three feet up the intestines, and then disappearing. Some had cicatrized, and some were cicatrizing. Both cases originated from ship fever in a very cleanly and healthy set of men; so that it seems as if the same poison might communicate to one ulceration, and to the other none."

Dr. Swett ingenuously added, "that his belief in the non-identity of the diseases lessened daily, and he could not discriminate between cases presenting intestinal lesions (typhoid), and those which did not (typhus)." Dr. Griscom, one of the physicians of the New York Hospital, states, that of ten autopsies of patients dying of ship fever in that institution, in July, August, and September, 1847, six presented follicular disease of the intestines. Peyer's plates were prominent in four, and ulcerated in two. Facts of this kind are so frequently observed in the New York Hospitals that they have, in a measure, ceased to attract the attention of many physicians once deeply interested in their investigation, in reference to the question of the identity of typhus and typhoid fever.

To the same conclusion tend the inquiries, relating to this subject, in other quarters of our country. A writer in the American Journal of the Medical Sciences, in speaking, it is presumed, of the ship fever in Philadelphia, says: "We have lately seen a great number of cases of fever in recently arrived Irish emigrants—the majority of the cases were well marked typhus fever; a few were of equally well marked typhoid fever; while in a third class of cases, the characteristics of these two fevers were so completely blended that it was very difficult to determine which of the two predominated." And he adds, "here then we have persons who have been exposed to precisely the same morbific causes, attacked with fever of a similar type, bearing the characters in some of the typhoid fever, in others of the typhus, and in others again a union, as it were, of the characteristics of both. This fact would seem to prove that the typhoid is a mere form or variety of the typhus fever." He also remarks, that "in the present state of our knowledge on this subject, it is, we conceive, much safer to consider the typhoid as one of the forms of typhus fever."

In view of the facts which have been stated, it seems to the committee that no reasonable doubt can remain that typhus and typhoid fever are identical. Were medical men united in this conclusion, might we not hope, that with undivided attention, and a union of effort more rapid advances would be made in determining the causes of the morbid condition of the Peyerian glands and other organs which occur in some cases of typhus and not in others? On this subject, we would, in conclusion, remark, that it appears to us, that the deposit of typhus matter, so called, occurs not only in the intestinal follicles and mesenteric glands, but also in and beneath the mucous membranes, in the lungs, spleen, and probably in the kidneys; that in some cases this form of epigenesis shows itself mostly, if not exclusively, in the intestines, in others in a different part, and in others again in various tissues; that in some instances it is not discoverable in any organ; that in the typhus of some seasons and countries it is generally present in the intestines, and in others, absent in many or a large majority of cases.—*Trans. of Am. Med. Association, Vol. I.*

